# Neuroimaging features of WOREE syndrome: a mini-review of the literature

**DOI:** 10.3389/fped.2023.1301166

**Published:** 2023-12-15

**Authors:** Laura Battaglia, Giovanna Scorrano, Rossana Spiaggia, Antonio Basile, Stefano Palmucci, Pietro Valerio Foti, Corrado Spatola, Michele Iacomino, Franco Marinangeli, Elisa Francia, Francesco Comisi, Antonio Corsello, Vincenzo Salpietro, Alessandro Vittori, Emanuele David

**Affiliations:** ^1^ Department of Medical Surgical Sciences and Advanced Technologies “GF Ingrassia”, University Hospital Policlinic “G. Rodolico-San Marco”, Catania, Italy; ^2^ Department of Biotechnological and Applied Clinical Sciences, University of L'Aquila, L'Aquila, Italy; ^3^ Unit of Medical Genetics, IRCCS Instituto Giannina Gaslini, Genoa, Italy; ^4^ Department of Anesthesia, Critical Care and Pain Therapy, University of L’aquila, L’aquila, Italy; ^5^ Department of Anesthesia and Critical Care, ARCO ROMA, Ospedale Pediatrico Bambino Gesù IRCCS, Rome, Italy; ^6^ Pediatrics Department, Ospedale Microcitemico, Cagliari, Italy; ^7^ Department of Pediatrics, University of Milan, Milan, Italy; ^8^ Department of Neuromuscular Disorders, UCL Queen Square Institute of Neurology, London, United Kingdom

**Keywords:** *WWOX*, developmental and epileptic encephalopathies (DEEs), WOREE syndrome, SDR domain, brain anomalies

## Abstract

The WWOX gene encodes a 414-amino-acid protein composed of two N-terminal WW domains and a C-terminal short-chain dehydrogenase/reductase (SDR) domain. WWOX protein is highly conserved among species and mainly expressed in the cerebellum, cerebral cortex, brain stem, thyroid, hypophysis, and reproductive organs. It plays a crucial role in the biology of the central nervous system, and it is involved in neuronal development, migration, and proliferation. Biallelic pathogenic variants in WWOX have been associated with an early infantile epileptic encephalopathy known as WOREE syndrome. Both missense and null variants have been described in affected patients, leading to a reduction in protein function and stability. The most severe WOREE phenotypes have been related to biallelic null/null variants, associated with the complete loss of function of the protein. All affected patients showed brain anomalies on magnetic resonance imaging (MRI), suggesting the pivotal role of WWOX protein in brain homeostasis and developmental processes. We provided a literature review, exploring both the clinical and radiological spectrum related to WWOX pathogenic variants, described to date. We focused on neuroradiological findings to better delineate the WOREE phenotype with diagnostic and prognostic implications.

## 1. Introduction

Developmental and epileptic encephalopathies (DEEs) are early-onset disorders characterized by multiple seizure types, widespread epileptic discharges at the electroencephalogram, and neurodevelopmental delay (1–4). In recent years, next-generation sequencing (NGS) technologies, including exome and genome studies, revealed an increased complexity underlying DEEs, with the involvement of several genes implicated, such as the *WWOX* gene. Biallelic pathogenic variants of *WWOX*, represented by homozygous and compound heterozygous variants, have been associated with an early infantile epileptic encephalopathy known as WOREE syndrome (MIM: 616211) (5–8). Individuals affected with WOREE syndrome presented early onset epilepsy, with refractoriness to antiseizure medications (ASMs), development impairment with psychomotor delay, spastic tetraplegia, inability to walk, non-verbal communication and other additional features, generally dying within the first year of life (1). Brain imaging typically showed variable anomalies, such as myelination impairment, corpus callosum thickness, white matter anomalies, and/or cerebral atrophy (1, 9).

In WOREE syndrome, *WWOX* pathogenic variants lead to a variable loss of function of WWOX protein, which is highly conserved across species and mainly expressed in the cerebellum, brain, thyroid, hypophysis, and reproductive organs (1, 10, 11). WWOX includes 414 amino acids with two WW domains at the N-terminal end and a central short-chain dehydrogenase/reductase (SDR) domain (1, 12). It plays a crucial role in the biology of the central nervous system and is involved in neuronal development, migration, and proliferation. Functional studies using human organoids and animal models attempted to explain WWOX properties, exploring the phenotype spectrum related to *WWOX* loss of function. Specifically, WWOX, mainly expressed in neurons, oligodendrocytes, and astrocytes, acts as a scaffold protein, modulating the cytoskeleton and homeostasis of its substrates (9, 11–14).

Interestingly, oligodendrocyte progenitors present a high level of WWOX protein, in contrast with mature myelinated oligodendrocytes, suggesting a crucial role of the protein in myelin biogenesis, and in differentiation of these cells. Indeed, there is evidence of its function in cellular lipidic homeostasis and of its interaction with proteins involved in myelin biogenesis such as SEC23IP, SCAMP3, VOPP1, and SIMPLE (1, 15, 16). Moreover, loss of function of *WWOX* leads to hypo-myelinization, with a potential role in multiple sclerosis pathogenesis (1). Interestingly, when microglia were treated with lipopolysaccharides, a *WWOX* overexpression occurred, with the following activation of the immune system. Moreover, WWOX modulates the NF-kB pathway, and its loss of function was related to astrogliosis, suggesting an additional role of WWOX in neuroinflammation and activation of the immune response (1, 17–21).

Furthermore, WWOX protein presents a focused expression in the medial entorhinal cortex, basolateral amygdala, and layer 5 of the frontal cortex, which are mostly involved in memory, learning mechanisms, perception, and emotion control. It was observed that the loss of function of the WWOX protein was related to Alzheimer's disease, temporal lobe epilepsy, cognitive impairment, and neuropsychiatric disorders such as autism spectrum disorder (22–27). Interestingly, the WWOX protein is mostly expressed in the cerebellar cortex and in specific cell types, such as basket cells and granule cells, and hypomorphic mutations of *WWOX* have been associated with cerebellar disorders such as Spinocerebellar Ataxia Type 12 (SCAR12) (MIM: 614322). *WWOX* pathogenic variants have also been related to sex differentiation disorders (DSD), according to their high expression in reproductive organs and their potential role in gonadotropin synthesis, gonad development, and steroid metabolism (8, 12, 28, 29).

Furthermore, *WWOX* loss of function has also been related to early infantile refractory epilepsies. Functional studies showed that -/- mice presented GABAergic interneuron impairment and decreased expression of GAD65/67, enzymes that synthesize GABA, with evidence of cortical hyperexcitability and epilepsy onset (21, 30, 31).

WWOX also manages several pathways such as Wnt/β-catenin and TGFβ/SMAD and its loss of function is associated with carcinogenesis progression (9, 32, 33).

This study aims to better characterize neuroradiological features in WOREE syndrome, collecting all brain imaging data described to date in the literature, to accurately define the most common signs of the disease, which could lead to the definitive diagnosis when it is clinically suspected.

## 2. Genetic findings

*WWOX* is located on chromosome 16q23.1-q23.2 and crosses the second most common fragile site (FRA16D) in the human genome, with potential genomic instability (1, 12, 34).

A genotype-phenotype correlation has been suggested for *WWOX*-related disorders (35, 36). Specifically, WWOX loss of function caused by biallelic null variants (i.e., frameshift, nonsense, donor/acceptor splice site, and deletion) have been associated with the most severe phenotypes, represented by WOREE syndrome, with early death before 2 years of age, prenatal cerebral anomalies, and eye malformations (36). Concurrently, hypo-morphic genotypes characterized by biallelic missense variants have been related to a milder phenotype, represented by SCAR12. This rare spinocerebellar ataxia has been described in two families affected with early onset epilepsy, neurodevelopmental delay, intellectual disability, and cerebellar ataxia (35). Learning disorders, dysarthria, nystagmus, and decreased reflexes in the upper and lower limbs have also been reported in patients with SCAR12 who presented a partial loss of function of WWOX protein (35). Concurrently, patients carrying both a null and a missense variant presented an intermediate phenotype, whereas heterozygous variants have been described in healthy controls.

Interestingly, WWOX has been recently involved in several neurological disorders such as autism spectrum disorder (ASD), multiple sclerosis (MS), and Alzheimer's Disease (35). Patients with Alzheimer's Disease presented decreased WWOX protein levels, compared to age-matched healthy controls (37, 38). Specifically, functional studies revealed that the microtubule-associated protein tau was hyper-phosphorylated after interaction with glycogen synthase kinase 3β (GSK-3β) in the affected hippocampi, leading to neurofibrillary tangles formation with consequent neuronal death. In this circumstance, WWOX concurrently prevented tau hyperphosphorylation and increased the affinity to microtubules of tau proteins, blocking neurofibrillary accumulation and related neurodegeneration processes (39). Furthermore, WWOX interacted with another protein, TPC6AΔ, involved in tau aggregation and Aβ generation, suggesting a protective role of WWOX protein in Alzheimer's Disease (35, 40).

Notably, it was also observed that WWOX protein was decreased in the chronic active lesions of patients with MS, compared to age-matched healthy controls (41). Specifically, *WWOX* has been identified as a susceptible gene in MS and 48 *WWOX* pathogenic variants have been detected in affected patients (42). Interestingly, the role of WWOX in grey matter impairment in MS was described, with a relevant involvement of the protein in the myelination process and oligodendrocyte differentiation (35).

Moreover, deletions and duplications affecting the *WWOX* gene have been found in patients with ASD, and copy number variations (CNVs) of WWOX have been reported in patients with milder ASD phenotypes (35). However, the emerging role of WWOX in the pathophysiology of neurological disorders should be better evaluated in functional studies.

Even though pathogenic variants affect WWOX protein uniformly, some mutational hotspots have been reported in the literature to date. Specifically, the p.Gln230Pro pathogenic variant affects the SRD domain and has been described both in homozygosity and in compound heterozygosity in eight cases overall (1). Nevertheless, how missense variants affecting the SRD domain impair WWOX catalytic activity has not been demonstrated yet.

Moreover, two mutations affecting Glycine 137, a pivotal residue of the coenzyme binding region, have been described. Additionally, biallelic mutations affecting glutamic acid 17 and serine 318 combined with deletion or other missense mutations have been reported. Recurrent deletions and duplications of exons 6–8 have also been described. They led to truncated proteins or unstable products that were quickly degraded (1).

Furthermore, Proline 47 presumably may represent a mutational hotspot. It is a highly conserved residue, with a pivotal role in the function of the first WW domain. Specifically, it was observed that pathogenic variants affecting this residue, located in the first WW domain, both made WWOX unable to interact with partner proteins and impaired WWOX translocation to the nuclear compartment (1, 43). Other potential hotspot mutations could be represented by recurrent nonsense variants affecting Arg54*, frameshift variants such as Asp58Alafs*3, His173Alafs*67, His173Ilefs*5, and Glu306Aspfs*21, and splice site variants such as c.173-1G>T, c.173-2A>G, c.409+1G>T, c.517-2A>G, and c.606-1G>A (1).

However, functional studies should be performed, and knock-in animal models could better clarify the impact of point mutations on WWOX structure and function. To date, mice models revealed that WWOX presented a different tissue expression during developmental stages (35). A significant expression was detected in peripheral nerves, brain stem, and spinal cord during the first stages of embryogenesis, whereas a decreased level was detected in the latest stages, and an increased level was detected after birth.

Moreover, *WWOX* knockout mice, which had null alleles, showed deep structural anomalies such as malformations, neuronal impairment and degeneration, heterotopia, and defects of the midline. They exhibited a severe phenotype consistent with patients affected with WOREE syndrome, carrying null variants (29, 44). The full clinical spectrum of -/- mice was characterized by growth retardation, hypoglycemia, hypolipidemia, impaired steroidogenesis, bone defects, refractory seizures, ataxia, severe motor incoordination, imbalance, and premature death by 2 to 3 weeks after birth. This presumably indicates a pivotal role of WWOX in the neurobiology of the central nervous system at different developmental stages (45).

Concerning CNVs, most of them are intragenic, with the involvement of genomic regions crossed by *WWOX,* and deletions are more prevalent than duplications (1). A chromosomal microarray, choosing a quantitative method to analyze the WWOX locus, should be performed in patients with clinical features suggestive of WOREE syndrome. Indeed, individuals with WOREE syndrome present a high prevalence of CNVs, small structural variants detected by a quantitative approach. Concurrently, when whole exome sequencing (WES) and targeted gene panel sequencing are performed first, CNV investigation should be carried out, due to deletions and duplications, which often affect the *WWOX* gene. Furthermore, RNA studies could be useful to investigate if single-nucleotide variants (SNVs) and/or missense variants impair mRNA stability and disrupt the pre-mRNA splicing (2).

## 3. Clinical findings

WOREE syndrome is a developmental and epileptic encephalopathy (DEE) characterized by early onset epilepsy, with refractoriness to antiseizure medications (ASMs), development impairment with psychomotor delay, spastic tetraplegia, no walking, non-verbal communication, and other additional features, with death in first years of life (1). To date, 84 patients have been reported in the literature with descriptions of the individual phenotypes. Early onset epilepsy represented the main feature in these patients and occurred with different semiology. Specifically, early infantile DEE, epilepsy of infancy with migrating focal seizures (EIMFS), and infantile epileptic spasms syndrome (IESS) have been reported (2). Seizures included generalized tonic, myoclonic, clonic, and tonic-clonic seizures, focal clonic seizures, infantile spasms, eyelid myoclonia, and status epilepticus with refractoriness to ASMs. In affected patients, EEG showed background slowing with profound epileptic discharges (2). Developmental impairment preceded epilepsy and included severe intellectual disability, early-onset spastic tetraplegia, hypotonia, non-verbal communication, absence of ambulation, psychomotor delay, and microcephaly.

Dysmorphic features have been reported in affected patients and included short stature, round face with full cheeks, short neck, facial hypotonia, hypertelorism, arched and bushy eyebrows, long eyelashes, epicanthic folds, bitemporal narrowing, low anterior hairline, broad nose, high forehead, depressed nasal bridge, gingival hypertrophy low-set, and large ears (2). Scoliosis/kyphosis, movement disorders, and feeding problems have also been described. Moreover, ophthalmic anomalies such as poor or no eye contact, retinal degeneration, and optic atrophy have been reported (1).

We found a genotype-phenotype correlation only about the mortality risk. Specifically, null/null variants are associated with a higher mortality risk than missense/null and missense/missense variants (2). Here, respiratory problems were the main cause of death, while other causes included SUDEP, status epilepticus, and obstructive cardiomyopathy (2, 5, 46–49).

## 4. Imaging findings

**Figure 1:**
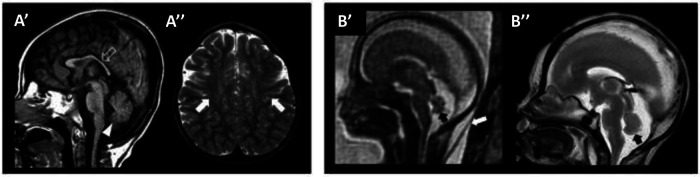
WOREE-associated neuroradiological features. A brain MRI of an affected patient was performed at 2.4 years of age (**A’**–**A’’**). (**A’**) The sagittal T1-weighted image reveals hypoplasia of the corpus callosum (empty arrow) and inferior cerebellar vermis (arrowhead). (**A”**) Axial T2-weighted images demonstrate mild atrophy of the frontal lobes with associated bilateral white matter hyperintensity (arrows). Fetal MRI (**B’**) and high-resolution post-mortem MRI (**B”**) of an affected patient were performed at 21 gestational weeks, demonstrating mild hypoplasia of the cerebellar vermis (black arrows). Note the slightly increased thickness of nuchal subcutaneous tissues on fetal MRI (white arrow). The laminar organization of the cerebral hemispheres and cortical gyration are appropriate for the gestational age (not shown). *Courtesy of Prof. Salpietro* (50).

One important step for the diagnosis of WOREE syndrome is based on brain imaging. Several studies identified MRI features related to *WWOX* gene mutations in children with epileptic encephalopathy and we will try to put together all the characteristics described to date, to define a unique radiological pattern (Figure 1, Table 1).

**Table 1:** MRI features of patients affected with WOREE syndrome reported to date.

MRI features	Abdel-Salam et al.	Tabarki et al.	Johannsen et al.	Piard et al.	He et al.	Banne et al.	Tarta-Arsene et al.	Iacomino et al.	Oliver et al.
Number of patients	1	5	2	20	1	56	1	2	13
Brain atrophy	1:1	5:5	2:2	11:20		+++	1:1		13:13
Severe frontotemporal atrophy	1:1								13:13
Hippocampal atrophy	1:1								7:13
Hypoplasia of the corpus callosum	1:1	5:5	2:2	15:20	1:1	+++	1:1	2:2	12:13
Myelination delay			1:2			+	1:1		6:13
Enlarged subarachnoid spaces	1:1			6:20	1:1				
Symmetric white matter hypersignal (T2)				3:20		+		2:2	10:13
Plagiocephaly and asymmetric lateral ventricle				2:20					
Circular lesions (hyposignal) of the medial part of the corpus callosum (T1)				1:20					
Optic atrophy						+++			13:13
Brainstem changes		1:5					1:1		2:13
Hypoplasia of the cerebellar vermis								2:2	
Progression of the abnormalities with age		5:5					1:1		7:13
Total number of patients	101								

MRI, magnetic resonance imaging; +, number of patients unspecified.

In 2014, Abdel-Salam et al. reported an affected girl born from consanguineous parents, who presented with growth retardation, microcephaly, epileptic seizures, retinopathy, and early death. WES revealed a nonsense homozygous mutation in *WWOX,* and brain MRI documented supratentorial atrophy with a simplified gyral pattern, hypoplasia of the hippocampus and the temporal lobe with consecutively widened subarachnoidal space, a thin hypoplastic corpus callosum, and hippocampal dysplasia with extracellular vacuoles in amygdala and hippocampus (47). Tabarki et al. then described five patients from two unrelated families who showed progressive microcephaly, early onset spasticity, refractory epilepsy, severe failure to thrive, and severe developmental delay. They carried the same homozygous mutation in *WWOX*. Interestingly, a brain MRI revealed a peculiar pattern of neurodegeneration, characterized by periventricular white matter volume loss, atrophy of the corpus callosum, flattening of the brainstem, and bilateral symmetrical lesions in the medial nuclei of the thalami in one patient. Of note, the cerebellum was not affected (51).

Furthermore, Tarta-Arsene et al. described in 2017 a similar brain MRI pattern in a boy with early-onset epilepsy, severe global developmental delay, persistent hypsarrhythmia at EEG, and epileptic spasms, carrying two *WWOX* mutations in a heterozygous state. Brain imaging was performed and documented a degenerative pattern characterized by cortico-subcortical atrophy, an extremely thin corpus callosum, delayed myelination, and flattening of the brainstem (52).

In 2018, Jessika Johannsen et al. examined two consanguineous patients with a homozygosity for the missense variant in the catalytic short-chain dehydrogenase/reductase (SDR) domain of the *WWOX* gene; both patients were characterized by early epilepsy refractory to treatment, progressive microcephaly, profound developmental delay, and brain MRI abnormalities. At the ages of 3, 5, and 21 months the imaging showed global atrophy, hypoplasia of the corpus callosum, and myelination delay with normal MR spectroscopy in patient 1. In patient 2, a cousin of patient 1, at the age of 3 and 6 months, brain imaging showed two similar features, global atrophy and hypoplasia of the corpus callosum (53). In the same year, Juliette Piard et al. studied 20 patients of 18 families with WOREE syndrome; they found a higher frequency of compound heterozygous mutations, consistently with a lower rate of consanguinity. The study detected abnormal brain MRI in 80% of them; specifically, 75% of patients were characterized by corpus callosum hypoplasia and 55% by cerebral atrophy. Other alterations included enlarged subarachnoid spaces, symmetric white matter hypersignal, plagiocephaly, asymmetric lateral ventricle, and circular lesions (hypo-signal) of the medial part of the corpus callosum (8). Another case of *WWOX* compound heterozygous mutations in a Chinese patient with WOREE syndrome was reported by Jing He et al. in 2019; in this case report, brain MRI revealed a widened subarachnoid space and a thin corpus callosum (54). Moreover, in 2020 an interesting study reported an abnormal cerebral cortex development in a family affected with neurodevelopmental impairment and refractory epilepsy, carrying a homozygous mutation in *WWOX*. Functional studies revealed a similar disorganization of cortical layers in mice, carrying a homozygous truncating *WWOX* mutation. Brain MRI of the two patients affected documented hypoplasia of the corpus callosum and mild atrophy of the frontal lobes with associated bilateral white matter hyperintensity. Notably, hypoplasia of cerebellar vermis related to WOREE syndrome was first reported in this study (50).

Similar features were found in 2021 by Banne et al., who retrospectively analyzed 45 variants in 56 WOREE published cases. A total of 34 out of 45 patients were characterized by loss of function mutations, and 11 were missense variants. Brain MRI imaging of children with WOREE syndrome showed abnormally thin or hypoplastic corpus callosum, progressive optic atrophy, and brain atrophy as the most common features, but in some cases, they also reported delayed myelination and white matter hyperintense signals (35). A recent study by Karen L. Oliver et al. in 2023 analyzed 13 patients from 12 families with WWOX developmental and epileptic encephalopathy (WWOX-DEE) due to biallelic pathogenic variants in WWOX. All of them showed severe frontotemporal atrophy. Brain MRI also demonstrated hippocampal atrophy, white matter signal abnormality, and volume loss with a very thin corpus callosum. Severe optic atrophy was also detected in all patients, whereas some patients presented brainstem changes, most of which were dorsal. Brain MRI revealed lesions in patient 1 and patient 7, at the age of 15 days and 19 days, respectively; specifically, patient 7 presented delayed myelination, a thin corpus callosum, and frontotemporal atrophy. Lastly, 7 of 13 patients underwent serial MRI, which showed progression of the abnormalities with age; specifically, related to the brain atrophy, which showed a change from mild to severe, and white matter alteration (2).

This review analyzed 101 cases of children with various mutations in the *WWOX* gene associated with WOREE syndrome from 2014 to 2023. Based on the literature data collected, hypoplasia of the corpus callosum and brain atrophy appeared to be the predominant MRI alterations related to this syndrome. Other relatively frequent features included symmetric white matter hyperintensity in T2-weighted images and optic atrophy. Furthermore, approximately 13% of the patients included in the study also demonstrated age-related progression. Atrophy of the hippocampus, delayed myelination, enlarged subarachnoid spaces, plagiocephaly, asymmetrical lateral ventricles, hypointense circular lesions in the medial part of the corpus callosum in T1-weighted images, brainstem alterations, and cerebellar vermis hypoplasia were observed in a lower percentage of cases, and therefore these findings seem to be less specific.

## 5. Discussion

Genetic neurologic disorders with neurodevelopmental delay and refractory epilepsy include a broad spectrum of monogenic conditions with expanding clinical differential diagnosis and genetic heterogeneity (55–60). Even in the era of next-generation sequencing (NGS), the etiology and disease mechanisms underlying these conditions remain unclear in a large number of cases (61–66). Defining the full spectrum of disease-causing molecular pathways underlying brain disorders is crucial to genetically diagnose patients with developmental and epileptic encephalopathies or delay and to assess potential personalized strategies for the follow-up and management of these affected children (67–70).

WOREE syndrome is a developmental and epileptic encephalopathy with a genetic etiology that affects the development and biology of the central nervous system. The loss of function of the *WWOX* gene leads to the absence of the protein encoded, associated with the highest mortality in affected patients. Nevertheless, the mechanistic effects of *WWOX* pathogenic variants on protein function are still not well known. Functional studies could better explain these aspects, allowing us to accurately characterize the disorder.

The most common neuroradiological findings were cerebral atrophy and white matter signal anomalies in the corpus callosum, with a myelination impairment. Interestingly, all affected patients presented brain anomalies at MRI, suggesting the crucial role of the WWOX protein in cerebral homeostasis and neuronal development. Recent mice studies showed how the loss of *WWOX* disrupts neuronal migration and CNS development across different species (50). However, organoids could better elucidate the impact of *WWOX* mutations on fetal developmental processes and neurogenesis (50).

Imaging data represent an essential instrument to best define phenotypes of WOREE syndrome, with diagnostic and prognostic implications, and they always should be detected in patients with relevant clinical features suggesting WOREE syndrome to better characterize the disorder. In patients with early infantile epileptic encephalopathy with refractoriness to ASMs and global developmental delay associated with characteristic neuroradiological patterns, the *WWOX* gene analysis should be included in the diagnostic workup.
